# Supplementary figures and images for: Adjudicating between face-coding models with individual-face fMRI responses
Source: PLoS Comput Biol. 2017 Jul 26;13(7):e1005604. doi: 10.1371/journal.pcbi.1005604 (PMC5550004; doi:10.1371/journal.pcbi.1005604)

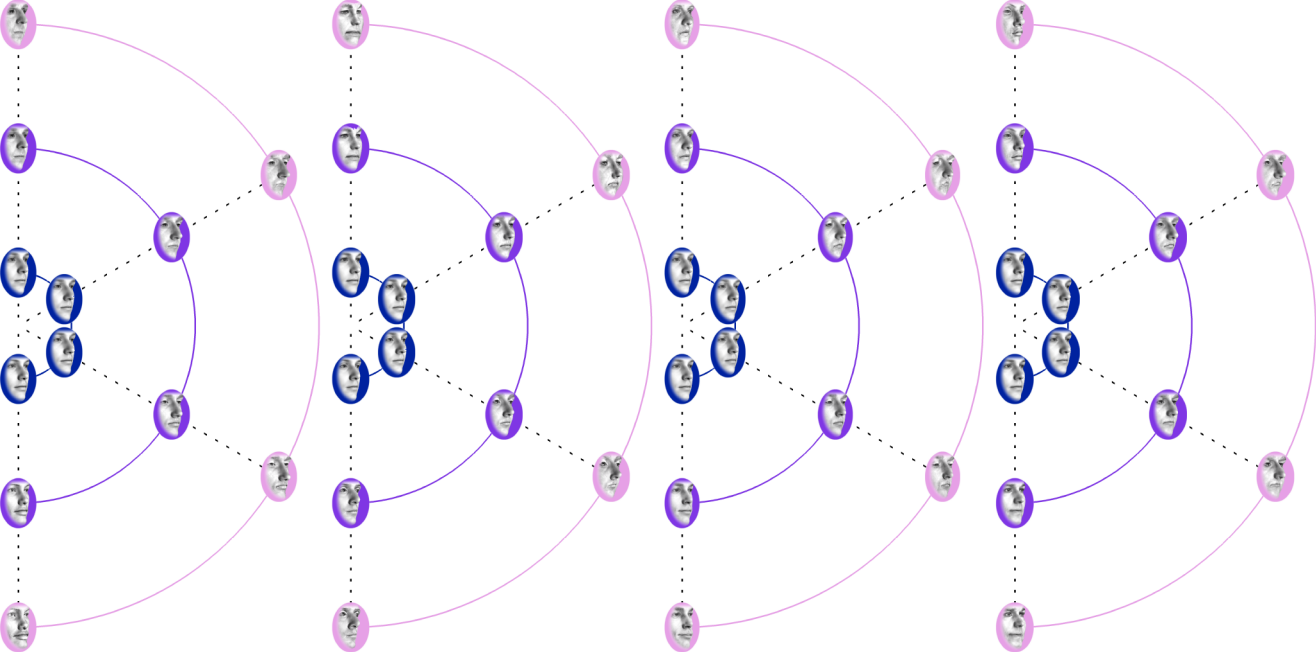

Supplement: S1 Fig — Each stimulus set shares the same underlying distance matrix in the reference PCA space, while the randomization of the orientation of the plane on which the faces are sampled ensures that each set is visually distinct. (PDF) [file pcbi.1005604.s001.pdf]

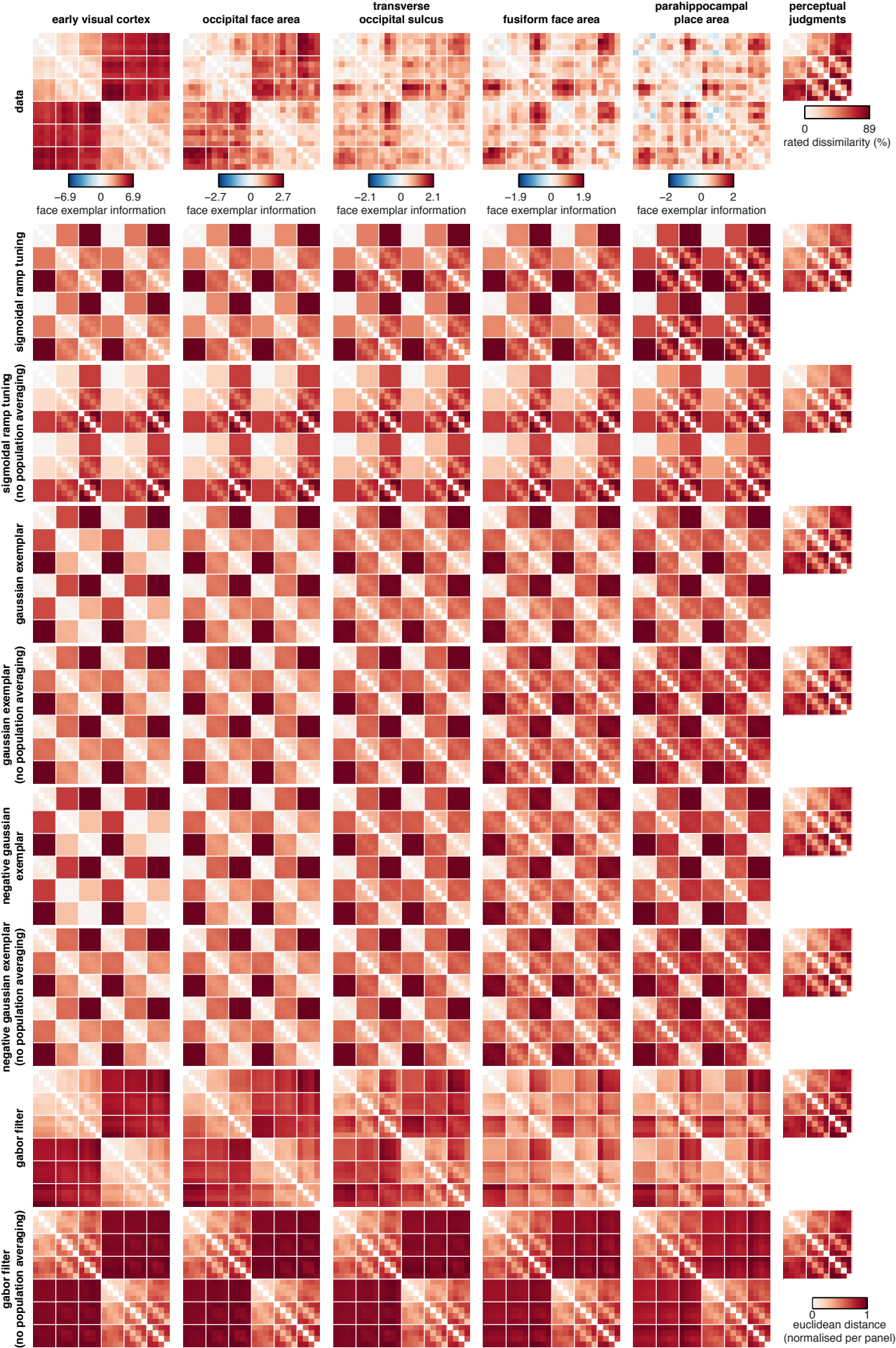

Supplement: S2 Fig — (PDF) [file pcbi.1005604.s002.pdf]

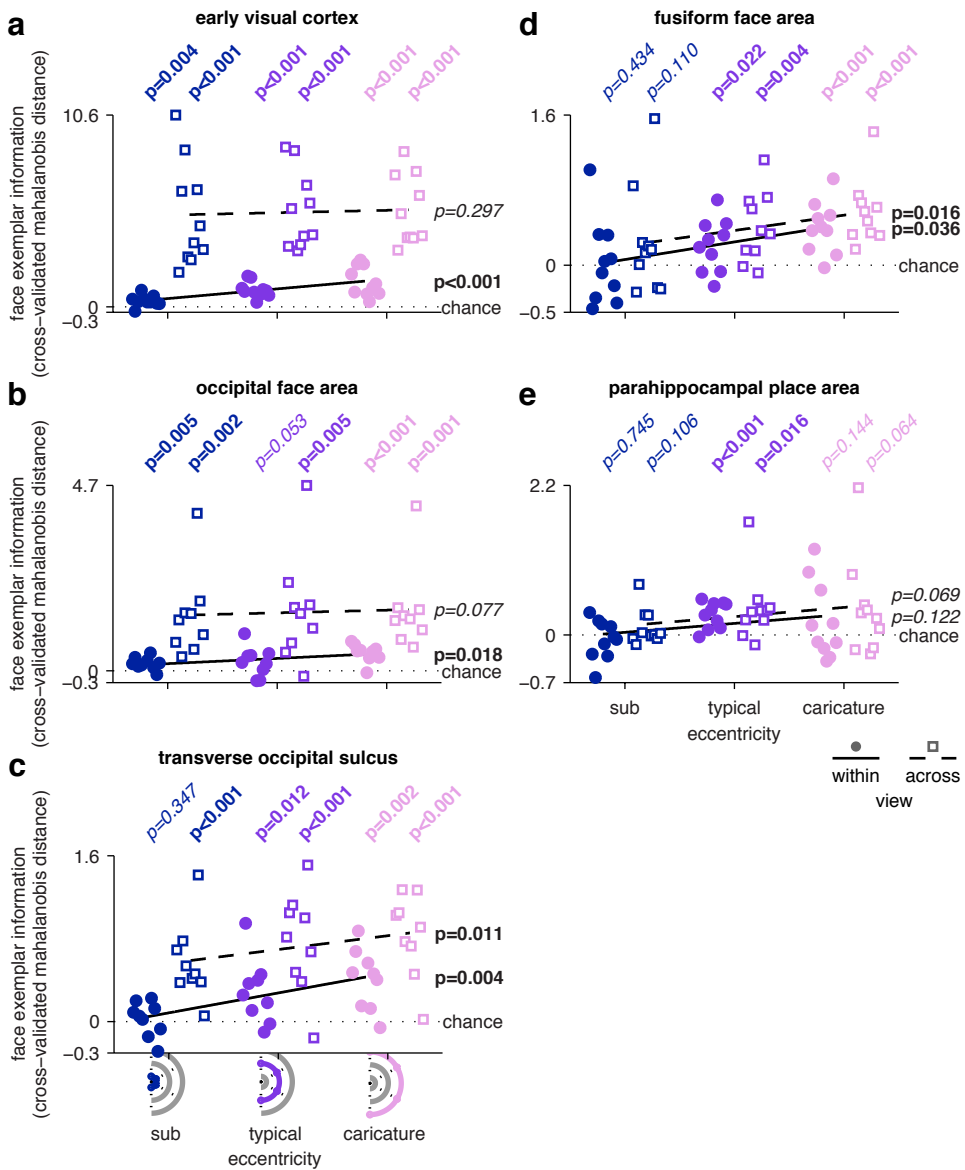

Supplement: S3 Fig — Each point reflects the mean performance for all directions at a given eccentricity level (4x4 block diagonals in Fig 1) for a single participant. Small random offsets have been added to each x coordinate for illustrative purposes, and a line shows the least-squares fit. Performance is plotted separately for distances within viewpoint (round markers, solid line, left offset) and across viewpoint (square markers, dashed line, right offset). All plotted p values are obtained through group analysis of single-participant estimates. Within viewpoint, cortical discrimination performance increases with eccentricity level in all regions except the parahippocampal place area (e). Across viewpoint, statistically significant effects are observed in the ventral temporal fusiform face area in the lateral temporal transverse occipital sulcus, but not in occipital areas (early visual cortex, occipital face area). (PDF) [file pcbi.1005604.s003.pdf]

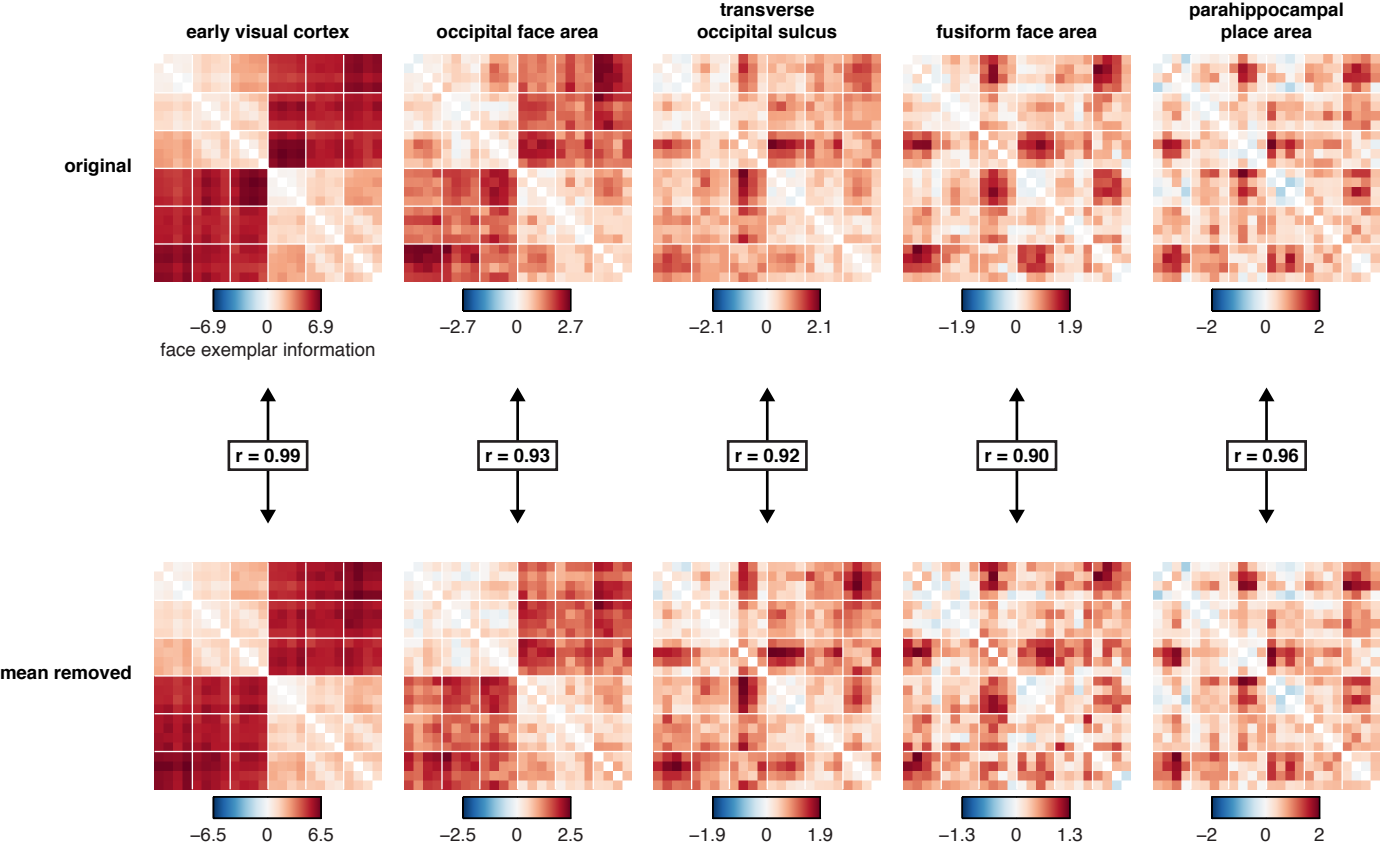

Supplement: S4 Fig — The top row shows original distance matrices, while the bottom row shows distance matrices after removing additive and multiplicative mean pattern effects (Materials and methods). The cited Pearson correlation coefficients are calculated at the group-average level. (PDF) [file pcbi.1005604.s004.pdf]

**a**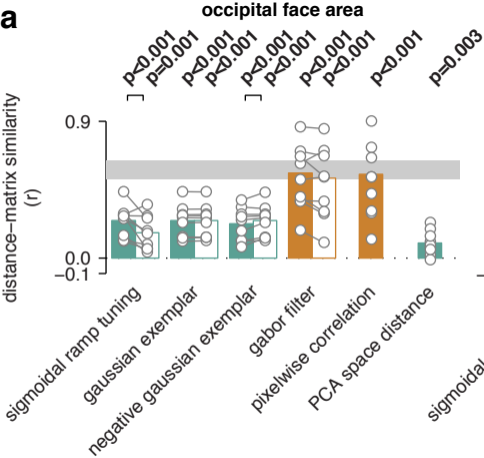**b**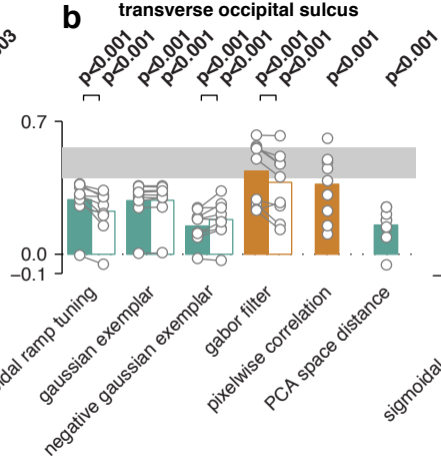**c**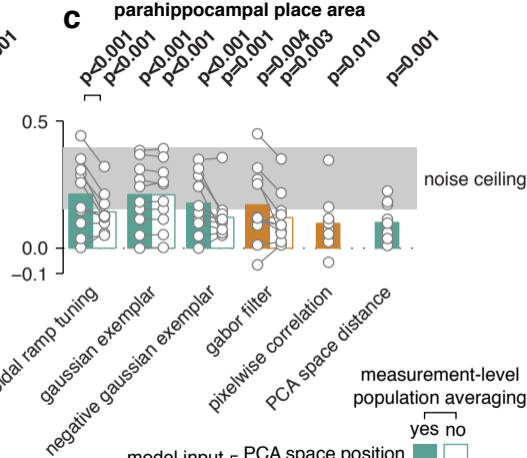

Supplement: S5 Fig — Plotted as Fig 5 in main text. (PDF) [file pcbi.1005604.s005.pdf]

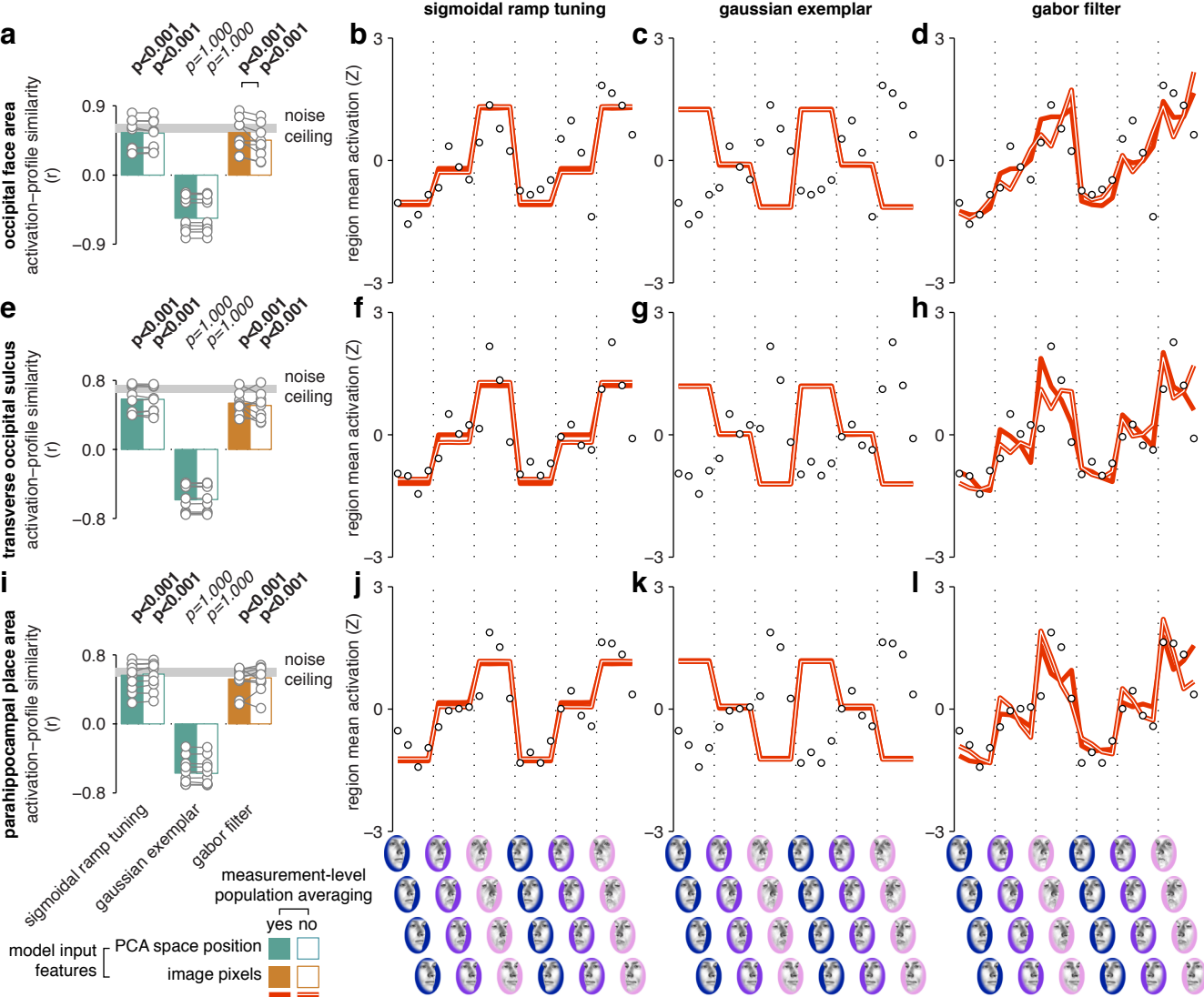

Supplement: S6 Fig — Plotted as in Fig 6 in main text. (PDF) [file pcbi.1005604.s006.pdf]

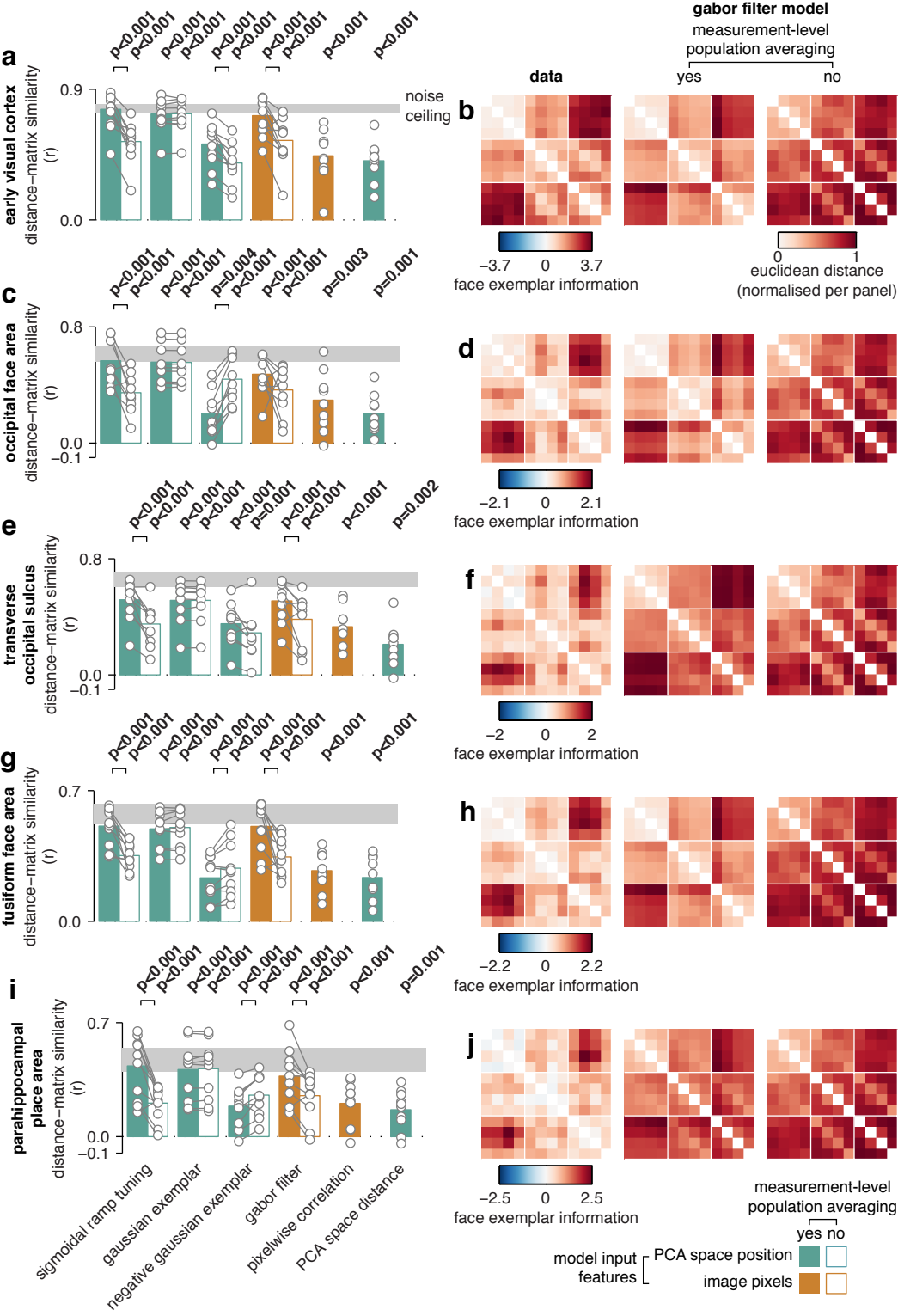

Supplement: S7 Fig — Plotted as in Figs 1 and 5 in main text. (PDF) [file pcbi.1005604.s007.pdf]

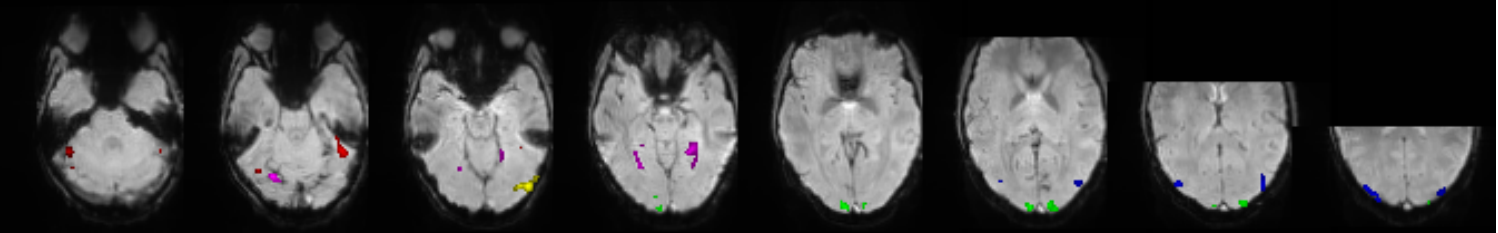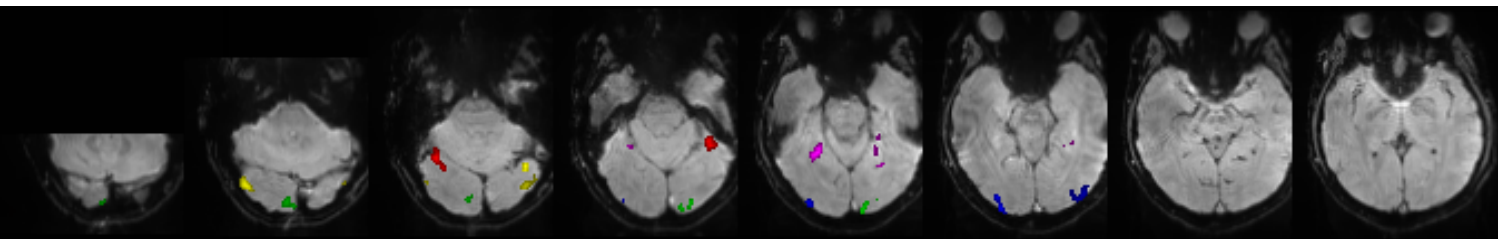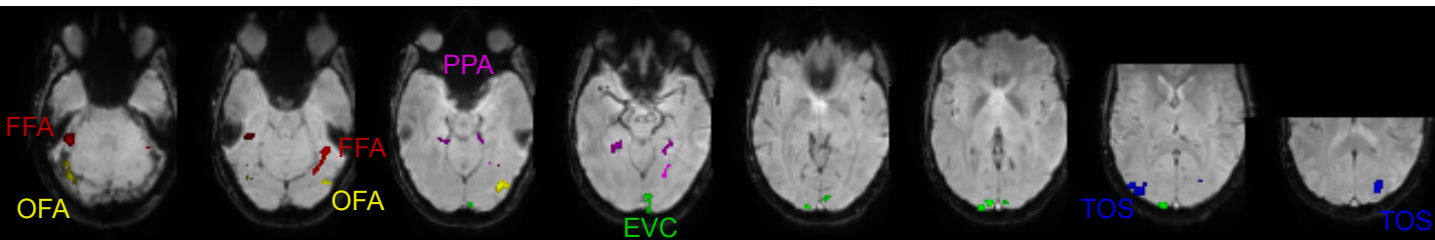

Supplement: S8 Fig — Regions are color-coded and labels are provided in the bottom row. Abbreviations and colors: EVC—early visual cortex, green; FFA—fusiform face area, red; OFA—occipital face area, yellow; PPA—parahippocampal place area, purple; TOS—transverse occipital sulcus, blue. (PDF) [file pcbi.1005604.s008.pdf]

rejection probability

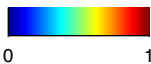

difference

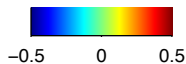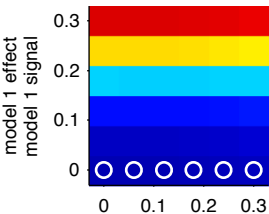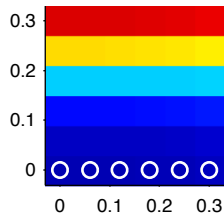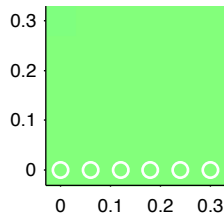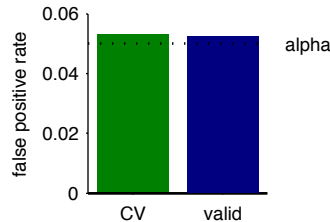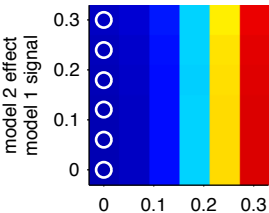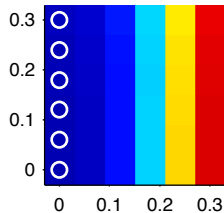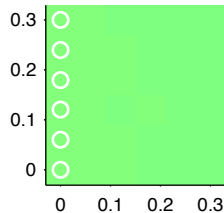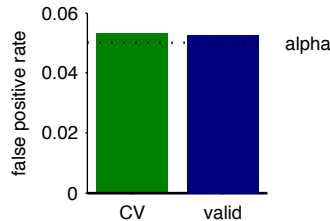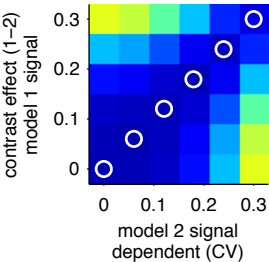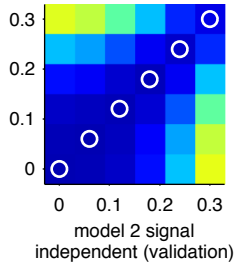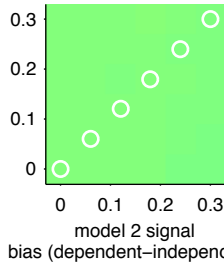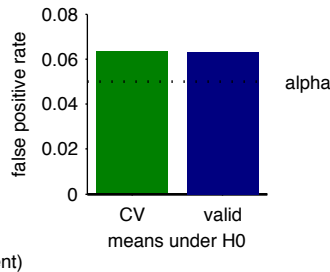

Supplement: S9 Fig — We simulated out-of-sample generalization performance for two arbitrary models, using methods that closely matched the ones described in this manuscript (for details, see S1 Code). We estimated potential bias by comparing cross-validated generalization performance (panels in leftmost column) with generalization to a withheld validation set (left column, subtraction in right column). The probability of rejecting the null hypothesis (p<0.05, T test) over 100000 simulations is plotted for tests of either model against zero (one-tailed test, first two rows of panels), and of zero difference between the models’ generalization performance (two-tailed test, bottom row). Each color-mapped image shows the rejection probability as a function of signal level for model 1 (vertical axis) and model 2 (horizontal axis). The null hypothesis case is highlighted with white circles. The bars in the rightmost panel summarize the mean rejection probabilities (ie, false positives) for each of these null cases. (PDF) [file pcbi.1005604.s009.pdf]

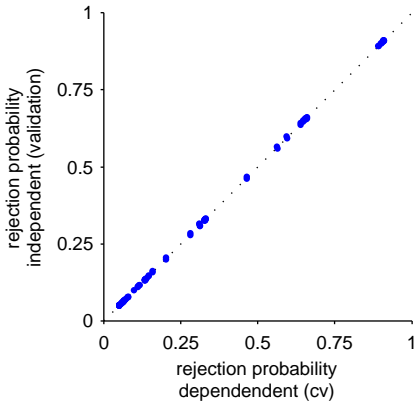

Supplement: S10 Fig — Each point represents the mean rejection probability for a unique set of simulation parameters (values in color-mapped images in S9 Fig). It can be seen that there is a close to unit relationship between rejection probability in the dependent, cross-validated case (vertical axis) and the independent, validation case (horizontal axis). (PDF) [file pcbi.1005604.s010.pdf]
